# Preschoolers' Dot Enumeration Abilities Are Markers of Their Arithmetic Competence

**DOI:** 10.1371/journal.pone.0094428

**Published:** 2014-04-08

**Authors:** Sarah A. Gray, Robert A. Reeve

**Affiliations:** Melbourne School of Psychological Sciences, University of Melbourne, Melbourne, Victoria, Australia; The University of Western Ontario, Canada

## Abstract

The abilities to enumerate small sets of items (e.g., dots) and to compare magnitudes are claimed to be indexes of core numerical competences that scaffold early math development. Insofar as this is correct, these abilities may be diagnostic markers of math competence in preschoolers. However, unlike magnitude comparison abilities, little research has examined preschoolers' ability to enumerate small sets, or its significance for emerging math abilities; which is surprising since dot enumeration is a marker of school-aged children's math competence. It is nevertheless possible that general cognitive functions (working memory, response inhibition in particular) are associated with preschoolers' math abilities and underlie nascent dot enumeration abilities. We investigated whether preschoolers' dot enumeration abilities predict their non-verbal arithmetic ability, over and above the influence of working memory and response inhibition. Two measures of dot enumeration ability were examined—inverse efficiency and paradigm specific (response time profiles) measures—to determine which has the better diagnostic utility as a marker of math competence. Seventy-eight 42-to-57 month-olds completed dot enumeration, working memory, response inhibition, and non-verbal addition and subtraction tasks. Dot enumeration efficiency predicted arithmetic ability over and above the influence of general cognitive functions. While dot enumeration efficiency was a better predictor of arithmetic ability than paradigm specific response time profiles; the response time profile displaying the smallest subitizing range and steepest subitizing slope, also displayed poor addition abilities, suggesting a weak subitizing profile may have diagnostic significance in preschoolers. Overall, the findings support the claim that dot enumeration abilities and general cognitive functions are markers of preschoolers' math ability.

## Introduction

A growing body of research has examined the neuro-cognitive bases of young children's math cognition. It has been claimed that the abilities to efficiently enumerate small sets of items and to compare magnitudes are indexes of core numerical competences that scaffold the acquisition of math abilities [1]–[3]. Insofar as this claim is correct, these abilities may be diagnostic markers of preschoolers' emerging math abilities and a basis for designing conceptually motivated intervention programs. However, unlike magnitude comparison abilities, little research has examined preschoolers' ability to enumerate small sets (e.g., dots – herein referred to as dot enumeration ability), or its significance for emerging math abilities.

Differences in preschoolers' magnitude comparison signatures (i.e., speed/accuracy of judging which of two quantities is more/less) are associated with their early math competence (e.g., scores on standardized math tests), even after other cognitive abilities are taken into account [4]–[8]. The relative dearth of research examining the significance of preschoolers' dot enumeration abilities as a marker of their emerging math abilities is surprising, given a growing body of research shows that dot enumeration abilities are a marker of school-age children's math competence, even when other numerical and/or general cognitive functions are taken into account [9]–[18]. Nevertheless, preschoolers' dot enumeration abilities are likely to be less well developed compared to those of school-aged children. It is possible that general cognitive functions (e.g., working memory, response inhibition) support preschoolers' emerging dot enumeration abilities, since these functions may scaffold early math processing [19]–[22]. To determine whether dot enumeration is indeed a core marker of emerging math competence, similar to magnitude comparison ability, it is necessary to establish whether preschoolers' dot enumeration abilities predict their emerging math abilities over and above the influence of general cognitive functions (working memory and response inhibition).

### Dot Enumeration

Dot enumeration tasks assess the speed and accuracy of enumerating sets of dots. A characteristic of these tasks is that small sets (n≤4) are enumerated accurately and rapidly, while larger sets (n≥4) are enumerated more slowly and performance is more error prone. The enumeration response time (RT) slope of small sets (known as the subitizing range) is relatively flat, while the slope for larger sets is steeper [15], [17], [23]. The discontinuity in dot enumeration performance over small and large sets is thought to reflect two distinct enumeration processes, namely a subitizing and a counting system [17].

The subitizing system may be supported by an ability to track small sets of objects in parallel that is apparent from infancy [2], [3], [24], [25]. For instance, infants are able to precisely track one, two, or three objects, but not four or more objects [24], [25]. The brain signatures (event-related potentials) of preverbal infants have also been shown to respond to the precise cardinal value of small but not large sets, when infants were viewing sets of dots in a number-alternation paradigm [2]. Findings also suggest that preschoolers' ability to process numerical information about small sets has developmental precedence over counting [26], [27]. Young preschoolers can associate number words with small sets (i.e., subitize) before they can reliably enumerate sets by counting [26], [27]. Moreover, two- to five-year-olds have been shown to accurately label the numerosity of small but not larger sets when arrays were displayed too briefly to allow counting, suggesting that preschoolers do not enumerate small sets by counting [27]. It has been proposed that the early ability to subitize is of developmental significance since it may provide a foundation for the development of important number concepts (e.g., cardinality, part-whole number relations) [1], [28]–[33].

Nevertheless, school-aged children's efficiency at enumerating sets more generally (small and large numerosities), is a marker of their math competence [16]. It is possible that the ability to efficiently process distinct numerosities, irrespective of subitizing ability, is important for supporting early math development [34], [35]. Little research has examined whether preschoolers' dot enumeration efficiency, or subitizing ability, is associated with emerging math competence.

### General Cognitive Functions

General cognitive functions, in particular working memory and response inhibition, have been associated with preschoolers' math abilities [19]–[22]. It is possible that working memory and/or response inhibition also support preschoolers' nascent dot enumeration abilities. Some researchers [36]–[38] but not all [39] have linked dot enumeration abilities in older children and adults with differences in working memory and aspects of inhibitory control [40], [41]. Specifically, it has been suggested that differences in dot enumeration abilities, in particular subitizing, may reflect differences in working memory capacity [36]. Inhibitory control may increase enumeration efficiency by inhibiting return to already enumerated items [40]; and for young children, response inhibition may play a role in inhibiting the desire to count or point to items in the subitizing range [42]. Preschoolers may be particularly reliant on these general cognitive functions to complete the dot enumeration task since their enumeration abilities are less well developed compared to older children. If preschoolers' dot enumeration abilities reflect aspects of working memory and/or response inhibition, then dot enumeration may not uniquely contribute to the prediction of emerging math competence over and above these general cognitive functions.

A growing number of studies with kindergarten and school-aged children suggest that dot enumeration ability, or the ability to process small sets [9], [10], and general cognitive functions, both contribute to the prediction of math competence [9], [10], [12], [18], [43], [44]. It has been proposed that general cognitive functions are important for supporting math learning because they help children to construct math skills from their domain-specific foundations [10], [44]. Working memory and response inhibition for instance, may assist young children in applying their numerical knowledge and skills to unfamiliar problems by helping them to maintain problem-specific information in memory, and inhibit distracting information [19]. General cognitive functions may be particularly important for supporting preschoolers' math processing, since their numerical skills are just emerging. Indeed, research shows that pre-frontal brain regions, often associated with executive functioning, are active in young children when acquiring math abilities, while math-specialised parietal regions become more active when math skills are more established [45]. It is possible that foundational numerical skills (i.e., dot enumeration) and general cognitive functions (working memory and response inhibition) both contribute to preschoolers' emerging math competence.

### Measuring Dot Enumeration Abilities

Two measures have been used to index school-aged children's dot enumeration abilities: inverse efficiency and dot enumeration paradigm specific measures. These measures reflect different aspects of dot enumeration performance. Inverse efficiency measures combine mean accuracy and RTs across all dot enumeration trials into a single measure of overall task efficiency [46]; these measures do not allow for an examination of differences in dot enumeration ability between the subitizing and counting ranges. Research has shown inverse efficiency measures to predict math competence in school children [16], [35], [47], [48]. Dot enumeration paradigm specific measures assess different components of dot enumeration RT signatures, including differences across the subitizing and counting ranges. Dot enumeration paradigm specific measures can include four RT parameters: the (1) subitizing range, (2) RT slope of the subitizing range, (3) y-intercept of the subitizing RT slope, and (4) RT slope of the counting range [15]. When all four parameters are considered in combination they allow for an assessment of children's unique dot enumeration RT profiles [15]. Reeve and colleagues [15] found that three over-arching dot enumeration RT profiles could be derived from a latent profile analysis of elementary school children's dot enumeration RTs, and these profiles were primarily distinguished by differences in subitizing parameters. Profile membership remained stable across the elementary school years and predicted computation performance longitudinally. The profile that exhibited the smallest subitizing range and largest subitizing slope and intercept consistently displayed the weakest computation ability. Indeed, deficits in dot enumeration subitizing signatures (i.e., smaller subitizing range and steeper RT subitizing slopes) have been found in children with developmental dyscalculia [13], [17], [35]. Compared to inverse efficiency measures, dot enumeration RT profiles may be more informative in identifying diagnostically-relevant information about underlying numerical processing deficits. Comparing the relationships between dot enumeration inverse efficiency scores and RT profiles and emerging math competence is important to establish which measure of ‘dot enumeration ability' has better diagnostic utility for preschoolers.

### Assessing Preschoolers' Math Abilities

Researchers have tended to use standardised measures to assess preschoolers' math ability [5], [21], which often involves aggregating scores across a range of math skills, some of which depend on formal learning experiences. Nevertheless, it is important to determine how core number abilities and general cognitive functions predict specific math skills [49]–[51]; since it is possible that different combinations of abilities contribute to the development of different math skills [9]. Non-verbal arithmetic abilities are thought to reflect the beginning of abstract numerical competence, and likely provide a foundation for later formal calculation abilities [34], [52], [53]. Huttenlocher and colleagues [54], [55], for example, found that non-verbal arithmetic develops from two-years of age and precedes verbal computation abilities. Nevertheless, there is also evidence to suggest that children master addition before subtraction [55]–[57], and young children may acquire subtraction knowledge independently from addition [57]. Non-verbal arithmetic will be assessed herein since it is an important component of preschoolers emerging math competence. Moreover, we examine preschoolers' addition and subtraction abilities separately, since these abilities may differ and be supported by different cognitive processes.

### The Current Study

In the present study we examine whether preschoolers' (42- to 57- month olds) dot enumeration ability is a diagnostic marker of their emerging math competence (non-verbal addition and subtraction) over and above the influence of working memory and response inhibition. We evaluate both efficiency (inverse efficiency scores) and paradigm specific (RT profiles) measures of dot enumeration to determine which measure is the better predictor of arithmetic ability. For both efficiency and paradigm specific measures of dot enumeration, we examine whether dot enumeration predicts non-verbal addition and subtraction abilities, taking into account working memory and response inhibition abilities. If preschoolers' dot enumeration abilities predict their non-verbal addition and subtraction performance, over and above the contribution of general cognitive functions, it would support the claim that dot enumeration ability is a diagnostic marker of emerging math competence.

## Methods

### Participants

Eighty preschoolers aged 42 to 57 months (*M* = 51.05 months, *SD* = 4.84), comprising 35 males and 43 females, participated. Children attended preschools in middle-class suburbs of an Australian city. In the Australian state in which testing was conducted, children attend preschool prior to starting formal schooling from 57 months of age (the minimum school starting age). The minimum age of participants in this study was 42 months: it is unlikely that children younger than this would have sufficient number knowledge to complete the dot enumeration task. Common to Australian preschools, the sample comprised children from diverse ethnic and cultural backgrounds; nevertheless, all children spoke English fluently. All participants had normal or corrected-to-normal vision, and none had reported learning difficulties.

### Ethics Statement

The study and consent procedures were approved by the Human Research Ethics Committee, University of Melbourne. Written consent to participate was obtained from children's parents/guardians. Due to their young age, children did not provide written consent, but their verbal consent was obtained prior to testing. Children who did not consent did not participate and no information was recorded for them.

### Materials and Procedure

Children individually completed (1) dot enumeration, (2) non-verbal addition and subtraction, (3) delayed alternation (working memory), and (4) Go/No-Go (response inhibition) tasks, in a quiet setting in their preschool. Tasks were completed in a random order over the course of a single day and took 15–20 mins to complete. The same interviewer tested all children. Prior to completing tasks, a counting pre-test (count list recitation) was conducted in which children were asked to count as high as they could, with an upper limit of 30. Only children who could count to at least five fluently in the pre-test participated in the study. The pre-test was conducted to ensure children who participated had some basic awareness of numbers. Two children were excluded on the basis of the pre-test, resulting in final sample of 78 children.

Stimuli for the dot enumeration and Go/No-Go tasks were presented on a laptop computer, using E-Prime (Version 2.0) software. Children sat approximately 30cm from the screen and stimuli were presented at eye-level. A hash mark appeared in the centre of the screen 1000ms prior to a to-be-judged target. In the dot enumeration task, similar to previous research [13]–[18], [35], targets remained on the screen until the child provided a response. In the Go/No-Go task, target arrays remained on the screen for a maximum of 1500 ms [58].

*Dot enumeration* stimuli comprised purple dots randomly arranged on a white background. Similar to previous research [12]–[18], [35], all dots were equated in size (2 cm in diameter) and numerosity (*n* = 1 to 5) varied across trials. Dots were positioned within a grid with an external perimeter of 15 cm by 11 cm. Individual dot positions for each array were selected pseudo-randomly with the constraint that the minimum distance between any two dots was 2 cm (to reduce an apparent clustering of dots). The maximum distance between two dots was 6.7 cm.

Dot numerosities one to five were selected because extensive pilot testing showed that all children in the age-range of interest knew number words up to five and were be able to give an accurate response for dots in this range. Our pilot testing also showed that few preschoolers could subitize beyond three dots and that one to five dots were adequate to assess enumeration skills beyond the subitizing range.

Children completed five practice trials, in which they were instructed to say as fast and accurately as possible how many dots appeared on the screen. In test trials, 1 to 5 dots were presented in a random order six times each, across two blocks of trials. The interviewer pressed a key to record children's RTs and noted their responses. The interviewer sat to one side of the computer and were able to see the child, but unable to see the computer screen or the number of dots displayed (similar to previous research [15]; a voice activated recording system was not used since children might occasionally count aloud and/or make other verbalisations).

Addition and subtraction abilities were assessed using Levine et al.'s [55] *non-verbal arithmetic* task. Variations of this task have been used to assess arithmetic abilities in young children [22], [59]–[61]. The task used herein comprised four addition and four subtraction trials (in the order, 2+1, 4+1, 3−1, 4−1, 1+3, 3−2, 2+2, and 4−2). A 30 cm×20 cm mat was placed on a table in front of the child and another in front of the interviewer. Ten 2^3^cm yellow blocks were in the centre of the table. For addition trials, the interviewer placed blocks comprising the augend on her mat, covered it with a third mat so the augend was no longer visible, and then took blocks comprising the addend, lined them up beside the mat, and slid them under the cover. Children were asked to “make your mat look just like mine”, which involved placing an equivalent number of blocks on their mat that corresponded to the interviewer's mat. After the child gave an answer, the interviewer lifted the cover to reveal the sum. For subtraction trials, blocks comprising the subtrahend were removed from the mat after it had been covered. No number words were used in the administration of the task. We analyse addition and subtraction problems separately as Levine et al. [55] found a tendency for 4-year-olds to be more successful solving non-verbal addition than subtraction problems, and as noted, it is possible that addition and subtraction abilities develop separately in young children.

Working memory was assessed using the *Delayed Alternation* task. This task is considered a measure of working memory because it requires children to update and maintain a mental representation of a rewards location across a filled delay, and to use this information to guide subsequent responses [62]. The task is ideal for preschoolers because it requires only simple responses, does not necessitate children to remember complex verbal instructions, draws on everyday experience and knowledge, and provides frequent rewards [20], [63]. Delayed alternation has been shown to be a reliable measure of working memory in typically developing preschoolers, and performance improves with age [63]. Moreover, preschoolers' performance on the delayed alternation task has been associated with their math ability [19], [64]. The delayed alternation task used herein comprised 16 trials. At the beginning of each trial, the experimenter asked the child to close their eyes, following which the experimenter hid a reward (a sticker) in one of two wells in a testing board. The wells were covered with identical inverted cups, and after 10 seconds the child was asked to open their eyes and decide which well contained the reward. If correct, the child could keep the reward, and on the next trial the reward location was alternated to the opposite well. If incorrect, the reward location did not alternate. Following Wiebe et al. [62], the maximum number of consecutive correct responses, minus the maximum number of consecutive incorrect responses were analysed since this measure is argued to conceptually represent children's ability to maintain task demands.

The *Cat-Mouse* version of the *Go/No-Go* task was adapted from Simpson and Riggs [58]. The Go/No-Go task is a widely used measure of inhibitory control across the lifespan and computerised versions of the task have been validated for children as young as 3-years [58], [65], [66]. Throughout the task, an image of a mouse (go trials) or a cat (no-go trials) appeared in the centre of the laptop screen. Children were instructed to press the spacebar when a mouse appeared (to “catch” the mice) and to press no button when a cat appeared. The task comprised six practice trials, followed by 45 test trials (30 go and 15 no-go trials). Trial order was randomised, except with the constraint that no more than two cat trials occurred consecutively. Children's mean accuracy on no-go trials was calculated as a measure of response inhibition.

### Analytic Approach

Dot enumeration inverse efficiency scores were calculated using the formula; median RT/accuracy [16], [35], [46], [48], [67], [68]. Median RT comprised the overall median RT for correct responses across all set sizes (1 to 5), while accuracy comprised children's proportion of correct responses across all trials. Smaller inverse efficiency scores indicate better task efficiency. Inverse efficiency scores also account for possible speed-accuracy trade-offs in children's responding [46]. Multiple linear regression analyses were conducted to examine whether dot enumeration inverse efficiency scores predict non-verbal addition and subtraction, when also taking into account working memory and response inhibition.

The dot enumeration task-specific effects of interest were the (1) subitizing range, (2) subitizing range RT slope, (3) subitizing RT slope y-intercept, and (4) counting range RT slope. The four parameters were derived from children's RT slope functions for median RTs (correct response only) across numerosities one to five. Typically, the subitizing and counting ranges can be represented by separate linear RT functions, with the point of discontinuity between these functions indicating an individual's subitizing limit. The point of discontinuity (and therefore the subitizing range) can be determined by the point at which the RT slope function changes from a linear to an exponential function [15]. We calculated each child's subitizing range (taken as the highest numerosity subitized) by establishing the point of discontinuity in their RT slope function. The remaining parameters were then calculated based on a child's unique subitizing range; the subitizing range RT slope and subitizing RT slope y-intercept were taken as the x coefficient and constant term of a child's subitizing range RT function, while the counting range (numerosities outside of the subitizing range) RT slope was taken as the x coefficient of a child's counting range RT function. For instance, if a child's subitizing range was 3, it can be assumed that their RT slope was characterised by a linear function across numerosities 1 to 3, and an exponential function across numerosities 1 to 4. This child's subitizing range RT slope and intercept would be calculated across RTs for numerosities 1 to 3 and their counting range slope across RTs for numerosities 3 to 5. Children who did not appear to subitize based on the RT slopes were assigned a subitizing range of 1.

Previous research has examined the relationship between individual dot enumeration parameters (e.g., subitizing slope) and children's math abilities [11], [13], [17], [35]; therefore, we examined correlations between the four RT parameters and preschoolers' arithmetic ability to determine whether further exploration into any individual parameter as a marker of emerging math competence was warranted. Nevertheless, we were primarily interested in examining dot enumeration RT profiles, as this approach may be a more meaningful way of assessing paradigm specific RT effects than examining the contribution of the four dot enumeration parameters separately; individually, the four dot enumeration RT parameters carry information about only one aspect of dot enumeration ability, whereas RT profiles allow for an assessment of children's ability across all aspects of the task, including subitizing and counting ability. Similar to Reeve et al [15], we used latent profile analysis (LPA) (Latent GOLD 4.5; [69]) to identify distinct dot enumeration RT profiles embedded within the overall RT distribution. Children's median RT's for correct responses to each numerosity tested (1 to 5) were entered into LPA (a total of five continuous variables). We were interested in identifying differences in children's RT patterns within and between dot enumeration set sizes, as patterns of change in RT with increasing set size carry important information about children's unique enumeration ability and their underlying number representations (e.g., their subitizing limit).

LPA identifies discrete profiles (subgroups) of individuals who share similar response patterns on a set of continuous variables via a probability-based classification [15], [69]. The probability of individuals belonging to each profile (the number of profiles differ according to the model being tested) is determined and individuals are classified into the profile for which the probability is highest [69], [70]. The numbers of profiles are not arbitrary or predetermined, but are determined post analysis, by comparing the goodness-of-fit statistics for a range of models that comprise different numbers of profiles. The goodness-of-fit statistics include the Bayesian Information Criterion (BIC), the Akaike Information Criterion (AIC), and the Consistent Akaike Information Criterion (CAIC). These criteria weigh the fit and parsimony of a model, with a lower value indicating a model that better fits the data. An R-squared value of entropy is also used to determine how well the model predicts profile memberships; entropy values closer to 1 indicate better predictions [69].

LPA is a more meaningful method of identifying ability subgroups than grouping children based on an atheoretical cut-point (e.g., median split). Further, LPA does not rely on any modelling assumptions (i.e., linear relationships, normal distributions, homogeneity), and therefore is also advantageous over traditional clustering techniques [15]. Therefore, LPA was considered the most appropriate method for answering our research question pertaining to the identification of distinct RT profiles embedded within preschoolers' dot enumeration RT distributions: for more detailed discussions on LPA see [15], [71]–[73].

Following the identification of dot enumeration RT profiles, we examined between-group differences in the four dot enumeration RT performance parameters (subitizing range, subitizing RT slope, subitizing RT slope y-intercept, and counting range RT slope). We then performed multiple linear regression analyses to examine whether dot enumeration profile membership predicted preschoolers' non-verbal addition and subtraction, over and above the influence of working memory and response inhibition.

## Results

Overall median dot enumeration response time (RT) and mean accuracy, as well as RTs and accuracy as a function of dot numerosity, are reported in Table 1. Consistent with previous practices only RTs for correct responses were analysed [15]. The mean overall dot enumeration inverse efficiency score (median RT/accuracy) was 2973.14 ms (*SD* = 1488.55). The mean subitizing range was 2.06 dots (*SD* = 0.83); the mean subitizing range RT slope was 363.01 ms (*SD* = 556.16); the mean subitizing range RT slope y-intercept was 1138.14 ms (*SD* = 709.71); and the mean counting range RT slope was 993.20 ms (*SD* = 702.46). The mean proportion of non-verbal addition and subtraction problems correct were 0.67 (*SD* = 0.29) and 0.63 (*SD* = 0.32) respectively. Mean working memory score was 5.69 (*SD* = 5.67) and mean response inhibition score was 0.87 (*SD* = 0.17). The mean highest number counted (taken from pre-test) was 18.14 (*SD* = 9.10). Table 2 displays correlations between each of these measures and children's age in months.

**Table 1:** Medians and SD's of Response Times and Mean and SD's of Accuracy on the Dot Enumeration Task, Overall and as a Function of Numerosity.

	Response Time		Accuracy	
Numerosity	*Median*	*SD*	*M*	*SD*
1	1472.00	505.99	1.00	.03
2	1938.50	765.60	.98	.08
3	2664.50	946.15	.91	.18
4	3407.50	1782.99	.79	.26
5	4390.00	1601.50	.62	.33
Overall	2290.50	925.84	.86	.13

**Table 2:** Zero Order Correlations.

	1.	2.	3.	4.	5.	6.	7.	8.	9.	10.
1. DE IE Score	1									
2. Subitizing Range	−.39**	1								
3. Subitizing Slope	.51**	−.50**	1							
4. Subitizing Intercept	.01	.34**	−.76**	1						
5. Counting Slope	.05	.43**	−.20	.24*	1					
6. Addition	−.56**	.16	−.11	−.12	−.01	1				
7. Subtraction	−.61**	.30**	−.19	−.11	.09	.57**	1			
8. Count Sequence	−.62**	.22	−.27*	−.02	−.04	.42**	.49**	1		
9. Working Memory	−.45**	.46**	−.25*	.10	.16	.38**	.53**	.41**	1	
10. Inhibition	−.29*	.12	−.24*	.09	.11	.34**	.28*	.33**	.24*	1
11. Age in Months	−.59**	.40**	−.38**	−.04	.14	.36**	.46**	.48**	.43**	.19

*p<.05. **p<.01.

*Note*. DE IE Score  =  Dot Enumeration Inverse Efficiency Score

### Dot Enumeration Efficiency, General Cognitive Functions, and Arithmetic

As shown in Table 2, preschoolers' dot enumeration inverse efficiency scores are correlated with their non-verbal addition and subtraction ability. Working memory, response inhibition, and age in months are also associated with addition and subtraction. Moreover, dot enumeration inverse efficiency scores are associated with working memory and response inhibition, and children's age in months. To investigate whether dot enumeration efficiency predicts preschoolers' non-verbal addition and subtraction, over and above the influence of working memory, and response inhibition, two separate multiple regression analyses were conducted. Children's age in months was also entered as a predictor to control for age-related differences. The highest number correctly counted in the count sequence recitation pre-test, was also included in the model to determine whether dot enumeration ability explained unique variance beyond that explained by any form of informal number knowledge.

The model predicting addition accuracy from dot enumeration inverse efficiency scores, working memory, response inhibition, age, and count sequence was significant (*F* (5, 72) = 8.475, *p*<.001) and explained 33% of the variance. An increase in dot enumeration inverse efficiency score significantly predicted a decrease in addition accuracy (β = −.428, *t* = −3.187, *p* = .002). Response inhibition performance was also a marginally significant predictor of addition, with increasing response inhibition scores predicting better addition accuracy (β = .174, *t* = 1.733, *p* = .087). Children's age in months, working memory, and counting abilities did not significantly contribute to the model predicting addition.

We were concerned that the relationship between dot enumeration inverse efficiency scores, response inhibition, and addition could be explained by general speed of responding to visual information; therefore, median RTs on Go trials of the Go/No-Go task were also included in the analysis as a proxy for basic response speed. RTs from Go trials (correct responses only) of the No-Go task have previously been used as a measure of processing speed in school-aged children and adults [74], [75], and preschoolers [76]. In a factor analysis study, McAuley and White [75] found Go trial RT to load on the same factor as other measures of processing speed, including a simple motor RT task, suggesting that it is a valid measure of processing speed across a broad age range (6- to 24-year-olds); although preschoolers were not tested. Herein, only Go trials that did not immediately follow a No-Go trial (*n* = 17 trials) were used to calculate median Go RTs in order to reduce the likelihood that response times were influenced by No-Go inhibition trials. We found no significant correlation between accuracy on no-go trials (inhibition) and correct Go trial median RTs (*r* (76) = .173, *p* = .130), suggesting that responses to these trial types were unrelated (i.e., faster RTs do not represent speed-accuracy trade-offs; [77]). When Go trial RT was included in the model predicting addition accuracy, the pattern of results did not change (*F* (6, 71) = 7.414, *p*<.001); both dot enumeration efficiency (β = −.429, *t* = −3.213, *p* = .002) and response inhibition (β = .221, *t* = 2.081, *p* = .041) remained significant contributors to the prediction of addition, albeit the predictive significance of response inhibition increased from the previous model.

The model predicting subtraction accuracy from dot enumeration inverse efficiency scores, working memory, response inhibition, age, and count sequence was also significant (*F* (5, 72) = 12.663, *p*<.001) and explained 43% of the variance. An increase in dot enumeration inverse efficiency score significantly predicted a decrease in subtraction accuracy (β = −.352, *t* = −2.849, *p* = .006), while an increase in working memory score significantly predicted an increase in subtraction accuracy (β = .287, *t* = 2.857, *p* = .006). No other variables significantly contributed to the prediction of subtraction performance. The pattern of results also did not change when Go trial RT was included in the model (*F* (6, 71) = 11.338, *p*<.001); both dot enumeration inverse efficiency (β = −.353, *t* = −2.902, *p* = .005) and working memory score (β = .240, *t* = 2.337, *p* = .022) remained significant contributors to the prediction of subtraction.

These analyses show that dot enumeration inverse efficiency scores explain unique variance in preschoolers' non-verbal addition and subtraction ability. Nevertheless, general cognitive functions are also related to preschoolers' non-verbal arithmetic ability (note: response inhibition contributed to the prediction of non-verbal addition and working memory contributed to the prediction of non-verbal subtraction). These patterns of findings were not accounted for by age in months, basic knowledge of number or basic RT (Go trial RT).

### Dot Enumeration Paradigm Specific Effects

Correlations between each of the dot enumeration RT parameters, and other variables, are shown in Table 2. Children's subitizing range RT slope, subitizing RT slope y-intercept, and counting RT slope (derived from their RT slope functions for median RTs, correct responses only, across numerosities one to five) were not significantly correlated with addition and subtraction performance (see Table 2), and therefore no analyses were conducted to further investigate the relationships between these measures. A small positive correlation was observed between subitizing range and subtraction accuracy. Nevertheless, a multiple regression analysis showed that subitizing range did not explain variance in subtraction performance over and above the influence of working memory, response inhibition, age in months, count sequence, and processing speed (Go trial RT) (for results, see Supporting Information S1).

It is possible that dot enumeration RT profiles may provide a more meaningful assessment of dot enumeration paradigm specific effects than examining the four dot enumeration parameters individually. Using latent profile analysis (LPA) of median RTs for each dot numerosity (1 to 5), we found that preschoolers could be classified as belonging to one of three dot enumeration RT profiles embedded within the overall RT distribution. We estimated models comprising one to six latent profiles, and as shown in Table 3, BIC and CAIC statistics were optimal for the 3-profile solution. Entropy values also suggested that the 3-profile solution predicted profile membership with higher precision than other solutions (see Table 3). Indeed, the best start seed was identical in replication analyses, which also suggests that a 3-profile solution was a robust representation of the data and did not represent a local maximum. In order to verify that the 3 profiles represented distinct and discontinuous groups, we calculated the average probability of children being classified into each of the three profiles. The average probability of children being classified into their profile of membership was very high (average probability = .984), while the average probability of children being classified into one of the other two profiles was very low (average probability = .007), providing evidence for the distinctness of the profiles.

**Table 3:** Fit Statistics for Models Comprising 1 to 6 Latent Profiles Obtained From a Latent Profile Analysis of Dot Enumeration Median Response Times on Numerosities 1 to 5.

Models	LL	BIC	AIC3	CAIC	N par	Entropy
1	−3188.68	6420.93	6407.36	6430.93	10	1.00
2	−3077.54	6246.57	6218.08	6267.57	21	0.88
3	−3011.23	6161.88	6118.46	6193.87	32	0.95
4	−2987.60	6162.53	6104.20	6205.53	43	0.91
5	−2977.29	6189.83	6116.57	6243.83	54	0.92
6	−2969.21	6221.60	6133.41	6286.60	65	0.91

*Note*. LL =  Log-Likelihood, BIC =  Bayesian Information Criterion, AIC3 =  Akaike's Information Criterion with a penalty factor of 3, CAIC =  Consistent Akaike Information Criterion, N par =  number of parameters, Entropy =  Entropy R-squared.

The three dot enumeration RT profiles identified by LPA comprised 27, 37 and 14 children and the profiles were labelled *Profile A, Profile B, and Profile C* respectively. Each profile's mean RT as a function of dot numerosity is shown in Table 4. A series of one-way ANOVAs showed that Profile C was slower identifying (naming) all numerosities compared to Profile A and Profile B, while the Profile B was also slower than Profile A on all numerosities (*p's*<.05, η^2^ ranged from .32 to .70). In terms of general dot enumeration RT patterns, Profile A, Profile B, and Profile C, exhibit fast, medium, and slow RT profiles respectively.

**Table 4:** Mean (SD) of Response Times (ms) as a Function of Dot Numerosity for the Three Dot Enumeration Response Time Profiles.

	Dot Enumeration Profile					
	Profile A (*n* = 27)		Profile B (*n* = 37)		Profile C (*n* = 14)	
Dot Numerosity	*M*	*SD*	*M*	*SD*	*M*	*SD*
1	1195.00	151.86	1498.54	265.61	2145.11	639.74
2	1246.81	169.11	1836.03	330.84	3029.21	787.60
3	1553.65	235.41	2643.38	575.90	3606.79	975.30
4	2284.41	586.10	3468.03	651.08	6026.18	2446.13
5	3212.30	782.05	4730.71	1769.99	5916.71	1203.26

We next characterised differences in dot enumeration ability between the three dot enumeration RT profiles based on the four dot enumeration RT parameters. Subgroup means on each of these parameters are shown in Table 5. An ANOVA revealed a significant between-group difference in subitizing range (*F* (2, 75) = 7.89, *p* = .001, η^2^ = .17). Profile A had a larger subitizing range than Profile B (*p* = .005) and Profile C (*p* = .002); the difference between Profile B and Profile C was not significant. Between group differences in subitizing range RT slopes were also found (*Welch's F* (2, 27.01) = 14.08, *p*<.001, η^2^ = .27—Welch's F is reported because of a violation in the homogeneity of variances assumption). Profile C had a steeper subitizing slope than Profile A (*p*<.001) and Profile B (*p* = .002), while Profile B also had a steeper subitizing slope than Profile A (*p* = .044). No between group differences in subitizing slope y-intercepts (*p* = .954), or counting range slopes (*p* = .359) were found. These findings show that different performance profiles are embedded within the overall RT distribution on the dot enumeration task, and that these profiles are best distinguished by their performance in the subitizing range (subitizing range and subitizing RT slope).

**Table 5:** Mean and Standard Error (SE) of the Four Dot Enumeration Response Time (RT) Parameters (Subitizing Range, Subitizing RT Slope, Subitizing RT Slope y-Intercept, and Counting RT Slope) for the Dot Enumeration Response Time Profiles; Profile A, Profile B, and Profile C.

	Dot Enumeration Profile					
RT Parameter	Profile A (*n* = 27)		Profile B (*n* = 37)		Profile C (*n* = 14)	
	*M*	*SE*	*M*	*SE*	*M*	*SE*
Subitizing range	2.52	0.14	1.89	0.12	1.64	0.23
Subitizing slope	68.59	22.60	372.27	67.92	906.32	244.03
Subitizing intercept	1121.94	43.01	1114.68	99.97	1231.38	363.33
Counting slope	857.58	109.95	1031.26	127.27	1154.15	196.42

### Dot Enumeration Profiles, General Cognitive Functions, and Arithmetic

To examine whether dot enumeration subgroup membership predicted non-verbal addition and subtraction when taking into account the influence of working memory and response inhibition, two multiple linear regressions were carried out. As profile membership was a categorical variable, it was dummy coded (dummy variable 1: 1 = Profile C, 0 = not Profile C; dummy variable 2: 1 = Profile A, 0 = not Profile A). Children's age in months and count sequence ability were again included as predictors in the regression models.

The model predicting addition accuracy from dot enumeration profile membership, working memory, response inhibition, age and count sequence was significant (*F* (6, 71) = 6.749, *p*<.001) and explained 30% of the variance. Membership in Profile C predicted a decrease in addition accuracy (β = −.300, *t* = −2.687, *p* = .009), while response inhibition score predicted an increase in addition accuracy (β = .253, *t* = 2.454, *p* = .017). No other predictors significantly contributed to the prediction of addition. The model predicting subtraction accuracy from dot enumeration profile membership, working memory, response inhibition, age and count sequence, was also significant (*F* (6, 71) = 8.593, *p*<.001) and explained 37% of the variance. Working memory score was the only significant predictor of subtraction, with increases in working memory predicting higher subtraction accuracy (β = .319, *t* = 3.041, *p* = .003). It should be noted that the patterns of results for both addition and subtraction did not change when we also entered children's median RT on Go trials of the No-Go task (as a proxy for basic RT) into the regression models (for results, see Supporting Information S2), showing that the results cannot be accounted for by differences in basic RT. The results indicate that only dot enumeration Profile C is a marker of preschoolers' arithmetic, but only for addition problems. Also, similar to the inverse efficiency analyses presented earlier, response inhibition and working memory remain predictors of addition and subtraction respectively.

## Discussion

The purpose of the research was to assess the value of preschoolers' dot enumeration ability as a core numerical marker of emerging math competence (non-verbal arithmetic). We assessed both dot enumeration efficiency (inverse efficiency scores) and paradigm specific RT measures (RT profiles) to determine which has greater diagnostic potential as a marker of preschoolers' math competence. The findings show that both dot enumeration efficiency and dot enumeration RT profiles predicted non-verbal arithmetic ability, over and above the contribution of working memory and response inhibition. Overall however, dot enumeration efficiency was a better predictor of emerging arithmetic competence than dot enumeration RT profiles, since only one profile (Profile C) was associated with arithmetic (addition trials only). Nevertheless, the finding that the RT profile characterised by a smaller subitizing range and steeper subitizing slope (Profile C) was associated with poorer addition performance is important, since it is similar to findings for school-aged children [15] and suggests that a weak subitizing profile may have potential as a diagnostic marker of emerging math difficulties (e.g., dyscalculia) in preschoolers. Working memory and response inhibition were also found to contribute to the predictions of preschoolers' non-verbal subtraction and addition abilities respectively. The findings show that both dot enumeration ability and general cognitive functions contribute to preschoolers' emerging math competence and have potential utility as diagnostic markers of emerging math (in)competence.

### Dot Enumeration Efficiency and Emerging Math Abilities

The current study is the first study to show that preschoolers' dot enumeration abilities (in particular, dot enumeration efficiency) is a marker of emerging arithmetic competence, over and above the influence of working memory and response inhibition. It seemed reasonable to expect that working memory and response inhibition would drive the relationship between preschoolers' dot enumeration and arithmetic abilities, based on the premise that young children might depend on these cognitive functions to support number processing, particularly while their number concepts and skills are being acquired [19]. Our findings suggest that preschoolers' dot enumeration abilities do not simply reflect differences in working memory and response inhibition. Nonetheless, the working memory and response inhibition measures used herein require the processing of non-numerical stimuli; an interesting aim of future research would be to investigate how number-specific working memory and response inhibition tasks are associated with dot enumeration and math abilities [78].

While general cognitive functions also contribute to predicting preschoolers' non-verbal arithmetic abilities, dot enumeration efficiency was the stronger marker. Dot enumeration efficiency has been shown to be a marker of school-aged children's formal math competence [16], [48]; we show that it is also a marker of non-verbal addition and subtraction abilities in young children. Previous research examining preschool predictors of math competence have largely focused on the role of non-symbolic magnitude comparison [4]–[8], [21]; however, magnitude comparison abilities have mixed utility in predicting math competence in older children [49]. In contrast, dot enumeration appears to have diagnostic utility for preschoolers and school children.

The findings also suggest that dot enumeration efficiency (inverse efficiency scores) is a better marker of preschoolers' math competence than paradigm specific effects (RT profiles). While dot enumeration efficiency was a strong predictor of non-verbal addition and subtraction, only one dot enumeration RT profile (Profile C) predicted addition (and not subtraction) ability, when also considering the influence of working memory and response inhibition. Research with older children has similarly shown that efficiency of processing numerical information (inverse efficiency scores) on tasks including dot enumeration and digit comparison, are better predictors of computation ability than corresponding paradigm specific effects (e.g., subitizing and counting range slopes on the dot enumeration task, and distance effects on the digit comparison task) [35], [47]. However, it should be noted that the latter studies examined individual dot enumeration RT parameters, rather than dot enumeration RT profiles. Herein we show that ability to quickly and accurately enumerate sets, irrespective of subitizing and counting abilities, has diagnostic relevance in preschoolers.

### Dot Enumeration Profiles and Emerging Math Abilities

Our findings also suggest that dot enumeration paradigm specific effects (RT profiles) may play a role in characterising children with emerging math difficulties. The three preschool dot enumeration RT profiles identified are similar to those identified by Reeve et al [15] in school-aged children; both studies identified a subgroup of children (Profile C) who displayed slower RTs, a smaller subitizing range, and steeper subitizing slope, compared to other children. In both studies, membership of this subgroup was associated with poorer arithmetic abilities (only non-verbal addition in the present study). Profile C characteristics are therefore associated with both informal and formal math difficulties, and it is possible that the subitizing difficulties of Profile C are an early marker of math learning difficulties associated with a core number deficit (i.e., dyscalculia). Indeed, several studies have shown weak dot enumeration subitizing signatures, including smaller subitizing range and steeper subitizing slope, to be a characteristic of school-aged children with dyscalculia [13], [17], [35]. Although dot enumeration efficiency scores may be better at predicting emerging math competence, lower dot enumeration efficiency can only indicate a learning delay, and cannot be used to characterise a numerical processing deficit; paradigm specific effects have the potential to provide additional diagnostically meaningful information that goes beyond the developmental delay perspective [35].

Nevertheless, our findings regarding the relationship between dot enumeration RT profiles and preschoolers' arithmetic competence differ from Reeve et al.'s [15] findings in one key respect; while we found that only Profile C predicted non-verbal addition, Reeve and colleagues found that all three dot enumeration profiles distinguished children's formal computation abilities. It is possible that dot enumeration RT profiles are less strongly associated with preschoolers' informal non-verbal arithmetic abilities than they are with school children's symbolic computation skills. It would be of interest to examine how preschool dot enumeration RT profiles are associated with emerging symbolic math abilities (e.g., counting abilities).

Further, compared to school children [15], preschoolers' dot enumeration abilities are just emerging and are characterised by greater variability in their responses. School-aged children likely process the dot enumeration task efficiently as their enumeration skills are more highly practiced; however, it is likely a novel task for preschoolers, and they may switch between counting and subitizing strategies to enumerate small sets [42]. As children get older their dot enumeration abilities (and RT signatures) become more stable and their RT profiles become more reliable markers of their math competence [15]. Nevertheless, while we obtained the conventional subitizing profiles, it is possible that limiting stimuli presentation times (and the potential to count in the subitizing range) may enhance differences in preschool subitizing RT profiles. In sum, our finding of three dot enumeration RT profiles that are similar to those found in school-aged children, and our finding that Profile C is associated with poorer non-verbal addition ability, suggests that preschool dot enumeration RT profiles (and in particular, subitizing RT signatures) may be diagnostically significant.

The ability to subitize is claimed to derive from an “innate” ability to precisely represent and track small numbers of objects [1]. How subitizing ability scaffolds math development nevertheless requires further examination. It is possible that subitizing may help children understand the concept of cardinality by enabling them to associate number words with distinct numerosities [26], [33]. When enumerating small sets and obtaining the same number as subitizing the set, children may associate counting and cardinality [27]–[29], [33]. Indeed, subitizing may support the development of enumeration ability more generally. Subitizing has also been suggested to facilitate an understanding of arithmetic concepts by allowing children to represent the effect of operations on small sets [29], [59]; for instance, subitizing allows children to see that adding or taking away objects from small sets changes the numerosity of the set. Given the potential diagnostic significance of subitizing for math development, it would be important to better understand how subitizing supports specific emerging math abilities. Moreover, if subitizing or dot enumeration ability more generally, does indeed support emerging math development, then interventions aimed at promoting these skills in preschoolers may have significant positive implications for their on-going math development.

### General Cognitive Functions

General cognitive functions (working memory and response inhibition) were found to contribute to prediction of preschoolers' non-verbal arithmetic ability, in addition to dot enumeration ability. This finding supports research associating working memory and response inhibition measures with a range of preschool math abilities [19]–[22]. It is possible that working memory assists performance on arithmetic tasks by allowing children to hold information in memory while new information is processed [78]. Response inhibition may facilitate arithmetic problem solving by allowing children to more efficiently identify relevant numerical information and ignore conflicting or distracting inputs [21]. Few studies that have examined the relationship between working memory, response inhibition, and preschoolers' math abilities have included domain-specific predictors, such as dot enumeration, in their models. The current study is the first to examine the relative contributions of dot enumeration, working memory, and response inhibition to preschool math ability and our findings support the claim that both domain-specific and general cognitive functions are important for emerging math development [9]. It is possible that working memory and response inhibition are particularly important for helping children to process math tasks while their numerical skills are nascent [19], [45], and that they become less important, relative to dot enumeration, as children get older and as their math processing becomes more efficient.

Early math skills may depend on different combinations of domain-specific and general cognitive functions [9]. This is consistent with our findings for addition and subtraction problems; specifically, working memory contributed to the prediction of subtraction, and response inhibition contributed to addition. These differences between addition and subtraction suggest that children may have approached these problems differently, and that different skills were required to support problem solving on the different problems. Moreover, this premise may also explain why dot enumeration RT Profile C was only associated with addition, and not subtraction. It is possible that children's conceptual and procedural understanding of subtraction was less well developed than that for addition [57]. Dot enumeration RT profiles may not have predicted subtraction differences because; firstly, dot enumeration profiles are less sensitive to differences in math competence than inverse efficiency scores, and secondly, general cognitive functions (working memory) may have influenced children's subtraction ability more than their addition ability, because the former problem type is less familiar. While it is not clear why working memory and response inhibition were associated with different arithmetic problem types (subtraction and addition respectively), it is evident that further research is needed to investigate differences in the addition and subtraction abilities in preschoolers. Indeed, the disparate findings for addition and subtraction also highlight the importance of examining specific math skills, rather than using standardised math measures that aggregate score across different math tasks [51].

## Conclusion

In summary, the findings of the present study suggest that dot enumeration abilities, like magnitude comparison abilities, are markers of preschoolers' emerging math competence and likely have diagnostic value. Indeed, dot enumeration is one component of Butterworth's [67] standardised *Dyscalculia Screener* (for children aged 6- to 14-years) and may also be useful as a screener of preschoolers math competence. Overall however, our findings suggest that research identifying markers of preschool math competence should not focus on the role of domain-specific or general cognitive abilities in isolation, as they both have an important role to play in identifying early math weaknesses.

## Supporting Information

Supporting Information S1 **Multiple regression analysis.** Multiple linear regression analysis predicting subtraction accuracy from subitizing range, working memory, response inhibition, count sequence, and basic RT.
(DOCX)

Supporting Information S2 **Multiple regression analyses.** Two multiple linear regression analyses predicting addition and subtraction accuracy from dot enumeration response time profiles, working memory, response inhibition, count sequence, and basic RT.
(DOCX)
