# Supplementary material for: Preschoolers' Dot Enumeration Abilities Are Markers of Their Arithmetic Competence
Source: PLoS One. 2014 Apr 8;9(4):e94428. doi: 10.1371/journal.pone.0094428 (PMC3979837; doi:10.1371/journal.pone.0094428)
Supplement: Supporting Information S1 — Multiple regression analysis. Multiple linear regression analysis predicting subtraction accuracy from subitizing range, working memory, response inhibition, count sequence, and basic RT. (DOCX) [file pone.0094428.s001.docx]

**Supporting Information S1: Multiple regression analysis.**

*Multiple linear regression analysis predicting subtraction accuracy from subitizing range, working memory, response inhibition, count sequence, and basic RT.*

A multiple linear regression was performed to predict subtraction accuracy from subitizing range, working memory, response inhibition, count sequence, and processing speed (Go trial median RT). The model was significant (*F* (6, 71) = 8.881, *p* < .001) and explained 38% of the variance in subtraction accuracy. Working memory was the only significant predictor, with working memory score predicting an increase in subtraction accuracy (β = .284, *t =* 2.502, *p* = .015).
