# Supplementary material for: Preschoolers' Dot Enumeration Abilities Are Markers of Their Arithmetic Competence
Source: PLoS One. 2014 Apr 8;9(4):e94428. doi: 10.1371/journal.pone.0094428 (PMC3979837; doi:10.1371/journal.pone.0094428)
Supplement: Supporting Information S2 — Multiple regression analyses. Two multiple linear regression analyses predicting addition and subtraction accuracy from dot enumeration response time profiles, working memory, response inhibition, count sequence, and basic RT. (DOCX) [file pone.0094428.s002.docx]

**Supporting Information S2: Multiple regression analyses.**

*Two multiple linear regression analyses predicting addition and subtraction accuracy from dot enumeration response time profiles, working memory, response inhibition, count sequence, and basic RT.*

A multiple linear regression was performed to predict addition accuracy from dot enumeration RT profile membership, working memory, response inhibition, count sequence, and processing speed (Go trial median RT). The model was significant (*F* (7, 70) = 6.522, *p* < .001) and explained 33% of the variance in addition accuracy. Belonging to dot enumeration Profile C predicted a decrease in addition accuracy (β = -.326, *t =* -2.954, *p* = .004), while response inhibition score predicted an increase in subtraction accuracy (β = .332, *t =* 3.038, *p* = .003). No other predictors significantly contributed to the explanation of addition accuracy.

A multiple linear regression was performed to predict subtraction accuracy from dot enumeration RT profile membership, working memory, response inhibition, count sequence, and processing speed (Go trial median RT). The model was significant (*F* (7, 70) = 7.873, *p* < .001) and explained 39% of the variance in subtraction accuracy. Working memory was the only significant predictor, with working memory score predicting an increase in subtraction accuracy (β = .276, *t =* 2.569, *p* = .012).
